# Making metallic glass design more intelligent by material networks

**DOI:** 10.1093/nsr/nwaf477

**Published:** 2025-11-04

**Authors:** Jun Shen

**Affiliations:** School of Materials Science and Engineering, Fujian University of Technology, China

Amorphous alloys, or metallic glasses, possess unique mechanical and functional properties due to their disordered atomic structure [1]. However, their discovery has historically relied on endless trial-and-error efforts, limiting efficiency and scalability. Recently, a research study by Hu *et al.* [2] introduces material networks—a data-driven strategy to accelerate the discovery of binary and ternary amorphous alloys. By representing the developed alloys as networks or graphs, the authors uncover hidden relationships between elements and predict novel alloy systems, bridging materials science with network theory and artificial intelligence.

The study begins by constructing high-level binary and ternary material networks. In the binary network, nodes represent elements, and edges denote experimentally confirmed binary glass-forming systems (94 edges, 38 nodes). The ternary network extends this concept, with nodes representing elements and triangles representing ternary systems (352 triangles, 47 nodes). The spatial layouts are optimized using the Fruchterman–Reingold algorithm, with nodes sized by atomic radii and colored by periodic table groups (see Fig. 1). This visualization reveals the complex interplay between elements in forming amorphous alloys.

**Figure 1.**
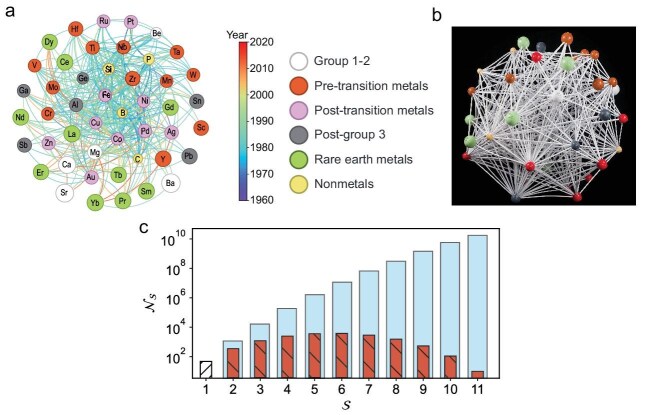
The material network of ternary metallic glasses. (a) Ternary network with historical information and elemental properties. (b) The 3D-printed ternary network. (c) Graph topology mining by clique analysis, illustrating the number of cliques with different number of nodes.

Figure 1 illustrates the material network of ternary metallic glasses, showing (a) the ternary network with historical information, (b) the 3D-printed ternary network, and (c) graph topology mining by clique analysis. A key innovation lies in the predictive power of the network clique analysis. Cliques, or fully connected subgraphs, reveal unexplored multi-component alloys. For instance, the binary network automatically forms higher-order cliques (e.g. triangles, rectangles), suggesting ternary and quaternary glass-formers. The ternary network exhibits cliques up to size $S = 11$, highlighting vast unexplored combinatorial spaces. This contrasts sharply with exhaustive combinatorial searches, demonstrating the network’s efficiency in prioritizing candidates. Some predicted multi-component alloy systems are shown in Table S1 in the online supplementary file, with a high-entropy metallic glass waiting for experimental confirmation. The authors also construct dynamic material networks to track the history of alloy discovery. By incorporating the earliest reporting year for each alloy, they

unveiled an innovation trap—researchers preferentially but unconsciously explored known element combinations after the 1980s, which led to stagnation in new system discoveries. This historical analysis underscores the importance of introducing new elements to break the innovation trap and drive forward breakthroughs.

Further, the ternary network classifies triangles into Auto, Fake, and Unknown categories. Auto triangles form automatically within the network topology, while Fake triangles lack one edge but represent potential alloys. Strikingly, ∼78% of real ternary alloys were embedded in Fake triangles in the binary network, validating the network’s predictive capability. This classification provides a low-cost experimental strategy for discovering new alloys.

The material networks exhibit abnormal scale-free behavior, with degree distributions following a power law relation. This deviates from classic scale-free networks [3] due to physical constraints from the limited number of elements in the periodic table. Elemental hubs (e.g. Al, Ni, Zr) show preferential attachment, attracting more connections during network growth. This behavior mirrors real-world networks like flight and communication systems, suggesting universal principles governing constrained network growth.

The implications of this work are profound. The network representation accelerates discovery by identifying Auto and Fake triangles, and even cliques, as experimental targets. It also inspires cross-network analysis, hinting at strategies for multi-component alloys. Beyond amorphous alloys, the framework applies to other materials (e.g. high-entropy alloys, ceramics) and integrates with AI-aided design. However, challenges remain, such as data scarcity [4] and the need for composition-specific predictions.

In conclusion, Hu *et al.* redefine materials discovery by translating amorphous alloys into interconnected networks. Their approach uncovers hidden design rules, predicts viable compositions, and mirrors real-world complex systems. By merging graph theory, materials science, and AI, this work lays the foundation for intelligent, data-driven alloy design. Important extensions to this work would use network analysis for the materials’ properties, such as relaxation dynamics [5], soft-magnetic properties [6], and possibly many others. More broadly, the ‘material network’ approach establishes a general paradigm to extract maximum knowledge from the precious experimental datasets, a common challenge across many sub-fields of materials science. This graph-based framework is readily transferable to other material systems where composition–property relationships are complex and data is scarce, such as high-entropy alloys, functional ceramics, and battery materials.

## Supplementary Material

nwaf477_Supplemental_File
