# Supplementary material for: Making metallic glass design more intelligent by material networks
Source: Natl Sci Rev. 2025 Nov 4;12(12):nwaf477. doi: 10.1093/nsr/nwaf477 (PMC12673570; doi:10.1093/nsr/nwaf477)
Supplement: nwaf477_Supplemental_File [file nwaf477_supplemental_file.pdf]

Table S1. Multi-component metallic glass prediction by ternary network cliques.

Alloy	Year	Reference
Al-Cu-Ni-Zr	2009	Acta Materialia 57, 1290 (2009)
Cu-Ni-Ti-Zr	2013	Journal of Materials Processing Technology 213, 2042 (2013)
Cu-Pd-Ti-Zr	2025	Journal of Alloys and Compounds 1010, 178081 (2025)
B-C-Fe-P-Si	2009	Journal of magnetism and magnetic materials 321, 2833 (2009)
Cu-Ni-Si-Ti-Zr	2006	Journal of Physics D: Applied Physics 39, 2600 (2006)
Al-Cu-Nb-Ni-Zr	2007	Materials Science and Engineering: A 457, 6 (2007)
B-C-Cr-Fe-Mo-P	2018	China Surface Engineering 32, 141 (2019)
B-C-Fe-Mo-P-Si	2025	Materials Today Advances 25, 100550 (2025)
Al-B-Cu-Hf-Ni-Si-Ti	2008	Journal of Alloys and Compounds 459, 251 (2008)
B-Co-Fe-Ge-Nb-Ni-Pd-Si-Ti-Zr		To be confirmed by experiments
